# Increased chitotriosidase 1 concentration following nusinersen treatment in spinal muscular atrophy

**DOI:** 10.1186/s13023-021-01961-8

**Published:** 2021-07-28

**Authors:** Maren Freigang, Petra Steinacker, Claudia Diana Wurster, Olivia Schreiber-Katz, Alma Osmanovic, Susanne Petri, Jan Christoph Koch, Kevin Rostásy, Björn Falkenburger, Albert Christian Ludolph, Markus Otto, Andreas Hermann, René Günther

**Affiliations:** 1 Department of Neurology, Technische Universität Dresden, Dresden, Germany; 2 Department of Neurology, Ulm University, Ulm, Germany; 3 Department of Neurology, Hannover Medical School, Hannover, Germany; 4 Department of Neurology, University Medicine Göttingen, Göttingen, Germany; 5 Department of Pediatric Neurology, Children’s Hospital Datteln, University Witten/Herdecke, Datteln, Germany; 6 German Center for Neurodegenerative Diseases (DZNE) Dresden, Dresden, Germany; 7 German Center for Neurodegenerative Diseases (DZNE) Ulm, Ulm, Germany; 8 Translational Neurodegeneration Section “Albrecht-Kossel”, Department of Neurology, University Medical Center Rostock, University of Rostock, Rostock, Germany; 9 German Center for Neurodegenerative Diseases (DZNE) Rostock/Greifswald, Rostock, Germany

**Keywords:** SMA, Chitotriosidase 1, Biomarker, Nusinersen, ASO

## Abstract

**Background:**

Studies regarding the impact of (neuro)inflammation and inflammatory response following repetitive, intrathecally administered antisense oligonucleotides (ASO) in 5q-associated spinal muscular atrophy (SMA) are sparse. Increased risk of hydrocephalus in untreated SMA patients and a marginal but significant increase of the serum/CSF albumin ratio (Qalb) with rare cases of communicating hydrocephalus during nusinersen treatment were reported, which confirms the unmet need of an inflammatory biomarker in SMA. The aim of this study was to investigate the (neuro)inflammatory marker chitotriosidase 1 (CHIT1) in SMA patients before and following the treatment with the ASO nusinersen.

**Methods:**

In this prospective, multicenter observational study, we studied CSF CHIT1 concentrations in 58 adult and 21 pediatric patients with SMA type 1, 2 or 3 before treatment initiation in comparison to age- and sex-matched controls and investigated its dynamics during nusinersen treatment. Concurrently, motor performance and disease severity were assessed.

**Results:**

CHIT1 concentrations were elevated in treatment-naïve SMA patients as compared to controls, but less pronounced than described for other neurodegenerative diseases such as amyotrophic lateral sclerosis. CHIT1 concentration did not correlate with disease severity and did not distinguish between clinical subtypes. CHIT1 concentration did show a significant increase during nusinersen treatment that was unrelated to the clinical response to nusinersen therapy.

**Conclusions:**

CHIT1 elevation in treatment-naïve SMA patients indicates the involvement of (neuro)inflammation in SMA. The lacking correlation of CHIT1 concentration with disease severity argues against its use as a marker of disease progression. The observed CHIT1 increase during nusinersen treatment may indicate an immune response-like, off-target reaction. Since antisense oligonucleotides are an establishing approach in the treatment of neurodegenerative diseases, this observation needs to be further evaluated.

**Supplementary Information:**

The online version contains supplementary material available at 10.1186/s13023-021-01961-8.

## Background

5q-associated spinal muscular atrophy (SMA) is a rare lower motor neuron disease caused by mutations in the *survival motor neuron 1* (*SMN1*) gene resulting in deficient biosynthesis of SMN protein, death of lower motor neurons, and consequently progressive muscle wasting. SMA is classified into clinical subtypes according to the best achieved motor milestone and age of onset [1]. In 2016, the United States Food and Drug Administration (FDA) approved the antisense oligonucleotide (ASO) nusinersen as the first disease-modifying drug for SMA for all patients, regardless of their age or disease stage, based on exceptionally convincing study results [2, 3]. In order to provide appropriate and standardized recommendations for choice of treatment and therapy (dis-)continuation, conclusive biomarkers are urgently needed [4–6].

Chitotriosidase 1 (CHIT1) is a human endochitinase, that is expressed by polymorphonuclear neutrophils and activated macrophages and is assumed to be involved in innate immune system responses, e.g. after allergen challenge or pathogen exposure [7–10]. Consequently, a 24 base pair duplication in the *CHIT1* gene (H allele) with high prevalence in European populations is associated with a deficiency in the activity of CHIT1 and is suspected to result in a higher susceptibility to infection [11]. CHIT1 hydrolyzes the β-(1-4)-linkage of *N*-acetyl-d-glucosamine, which is present in chitin chains [7, 8, 10]. Although chitin is not expressed in human cells, CHIT1 levels are elevated in serum and cerebrospinal fluid (CSF) in various diseases including Gaucher disease, idiopathic pulmonary fibrosis, sarcoidosis, chronic obstructive pulmonary disease and neurodegenerative/-inflammatory diseases such as Alzheimer’s disease (AD), frontotemporal dementia, multiple sclerosis (MS) and amyotrophic lateral sclerosis (ALS) [7, 12–24]. In patients with MS, the concentration of CHIT1 was elevated compared to controls and was found to be associated with other well-known MS-specific findings such as oligoclonal bands in CSF, elevated immunoglobulin G index, elevated CSF leukocyte count, and magnetic resonance imaging abnormalities showing dissemination in space, thus assuming to possess prognostic value [17]. In fact, CHIT1 activity is already used for the evaluation of response to immunomodulatory treatment in MS as a marker of inflammatory activity [25, 26]. In the CSF of patients with AD, CHIT1 activity was shown to be increased [24]. CHIT1 activity was discussed to be either a DNA damage marker and / or a response to chitin-like polysaccharides, which were found to accumulate as part of amyloid deposits in the brain of patients with AD, presumably as a consequence of impaired glucose metabolism [27, 28]. Patients with neurodegenerative dementia revealed significantly increased CHIT1 levels, which illustrates neuroinflammation as a common pathophysiological mechanism. However, because of overlapping levels of CHIT1 in prion disease, AD and frontotemporal lobar degeneration (FTLD), it is of limited diagnostic value [13, 29]. In ALS, CHIT1 levels were remarkably increased compared to both healthy and disease controls, and correlated with disease progression and severity. CHIT1 staining was restricted to specific areas along the spinal tract and was colocalized with markers of microglia and macrophages indicating the presence of microgliosis, which could not be detected in controls, AD or Creutzfeldt-Jakob disease. Additionally, CHIT1 levels were found to be higher in TDP-43 associated FTLD with ALS pathology compared to TDP-43 associated FTLD without ALS pathology, which implies a relationship of CHIT1 increase with a specific type of microgliosis/astrogliosis in corticoefferent pathways and/or association fibers [13, 20]. Further, CHIT1 levels were found to show the most extensive increase between the late presymptomatic and early symptomatic phases of disease, while patients after symptom onset present minimally increasing levels. CHIT1 levels of asymptomatic gene carriers did not differ from controls [15].

In SMA, recent studies suggest that microglial activation, driven by SMN protein deficiency, contributes to the phenotype of SMA and even precedes motor neuron loss [30]. Motor neurons were colocalized with an increased number of microglial cells in SMA mice which indicates a certain degree of neuroinflammation [31]. Increased risk of hydrocephalus in untreated SMA patients and a marginal but significant increase of the serum/CSF albumin ratio (Qalb) with rare cases of communicating hydrocephalus during treatment with the ASO nusinersen were reported [32–35].

The aim of this study was to evaluate CHIT1 as a marker of neuroinflammation in treatment-naïve patients with SMA and to investigate its dynamics during nusinersen treatment.

## Methods

### Standard protocol approvals, registrations, and patient consents

58 adult patients and 21 children with genetically confirmed 5q-associated SMA from 4 German motor neuron disease specialist care centers (Departments of Neurology in Dresden, Ulm, Hannover and Göttingen) and 30 age- and sex-matched controls were prospectively included in this study between 2017 and 2020. The local ethics committees of all participating sites approved the study and all patients signed written informed consent.

The demographic and clinical data of patients were collected including age, gender, disease onset, baseline weight and height, clinical subtype, number of *SMN2* copies if available and ambulatory status. Additionally, the need of CT-guided puncture and the use of traumatic or atraumatic puncture needle was recorded.

Patients received nusinersen treatment according to the prescribing information for up to 14 months.

CSF was obtained by lumbar puncture (LP), which was performed for intrathecal administration of nusinersen and was tested for total protein level, Qalb and cell count in the context of clinical routine by the in-house laboratory department of each participating center. As part of the clinical routine, CSF was examined microscopically for unusual cell types or altered cells within the cohort of the research site Dresden.

The samples designated for CHIT1 assay were stored at − 80 °C within 2 h after centrifugation (5 min; 6500 rpm). In total, 214 CSF samples were analyzed for CHIT1 concentration at three time points (T1 = baseline, T2 = 6.2 ± 0.6 months, T3 = 14.2 ± 0.9 months) using ELISA kits (CircuLex Human Chitotriosidase ELISA Kit, CY-8074, MBL, Belgium) at 1:10 dilution according to the instructions of the manufacturer. For quality control, a single CSF sample was run four times per plate for CHIT1. The mean intra-assay and inter-assay coefficients of variation were < 15%. At baseline, the CSF sample of one patient was insufficient for CHIT1 determination and consequently, this patient was excluded from the analysis.

To monitor motor and functional outcome, established motor scores (Hammersmith Functional Motor Scale Expanded—HFMSE [36], Revised Upper Limb Module—RULM [37]) as well as the revised ALS-Functional Rating Scale (ALSFRS-R) [38] were assessed concurrently at each visit. Motor scores comprise several items rating different motor skills with higher scores indicating better function. Ratings were performed according to the manuals.

### Statistical analysis

Statistical analysis and data visualization were performed using SPSS Statistics 27 (IBM, Chicago (IL), USA) and GraphPad Prism 5 (GraphPad Software Inc., San Diego (CA), USA). Unless otherwise stated, CHIT1 data and the assessed scores are presented as median ± interquartile range (IQR). CHIT1 data were not normally distributed as tested by Shapiro–Wilk test. We therefore applied rank-based, non-parametric tests for the baseline analysis. To estimate the comparability of study group and control group, we used Pearson's Chi-squared test for equal distribution regarding sex and Mann–Whitney U test concerning conformity of age. To compare CHIT1 levels of diseased individuals with controls, we calculated Mann–Whitney U test. To investigate the meaning of CHIT1 values for disease severity, we correlated CHIT1 baseline values with demographic features and clinical assessments using Spearman's rank correlation coefficient (*ρ*). Due to the significant association with height, we considered it a confounding factor and corrected for baseline height by partial correlation. A correlation coefficient of *ρ* < 0.3 was considered as a weak, *ρ* = 0.3–0.59 as a moderate, and *ρ* > 0.6 as a strong correlation (modified from [39]). We used one-way analysis of covariance (ANCOVA) with post-hoc Bonferroni adjustment for comparison of CHIT1 (dependent variable) between different patient subgroups considering height as covariate. To meet the assumptions of ANCOVA, we applied log transformation (decadic logarithm) to CHIT1 data. For longitudinal analysis under nusinersen treatment, we performed Wilcoxon signed-rank test to include all available data (n = 58) for the comparison between baseline and 14-month follow-up (representing third maintenance dose). Data sets with missing values were excluded pairwise for cross-sectional and longitudinal analysis. To comprehensively investigate CHIT1 levels over the treatment course, we used the Friedman test with post-hoc Dunn–Bonferroni adjustment after listwise exclusion in case of missing data. We performed standard multiple regression to determine the contribution of patient's height to CHIT1 change compared to other variables. For that purpose, we applied Johnson transformation to the difference of CHIT1 concentration between 14 months and baseline to approximate a standard distribution. Critical value was set as *p* < 0.05 two-sided. Whenever CHIT1 values were below the lower limit of quantification (e.g. for 7.6% in all disease samples; 15.2% in baseline disease samples), we used the lower limit of quantification as value in order not to exclude these measurements from the analysis.

## Results

58 adult patients and 21 children with SMA type 1 (n = 7), type 2 (n = 33) or type 3 (n = 39) were included in the analysis. Median age was 31 years (IQR 17–43), 52% were female. The control group was age- and sex-matched and comprised 23 adults and 7 children without suspected neurodegenerative or neuroinflammatory disease (healthy controls: n = 23, normal pressure hydrocephalus: n = 3, idiopathic Bell’s palsy: n = 4). In the control group, median age was 30 years (IQR 17–44), 60% were female. The distribution of sex did not differ significantly between the groups.

**Fig. 1:**
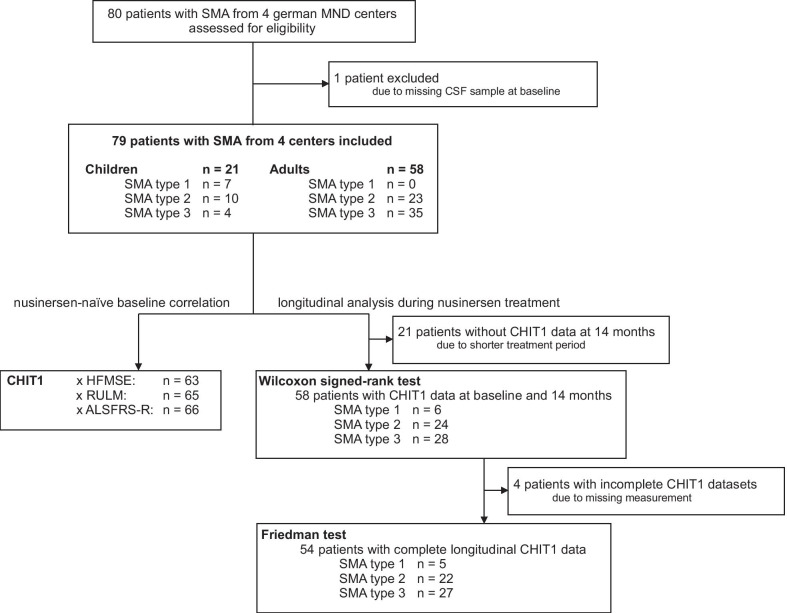
Study profile

Details of study group characteristics and study profile are presented in Table 1, Additional file 1: Table S1 and Fig. 1.

**Table 1:** Study group characteristics

	SMA (n = 79)	Controls (n = 30)
Age (year), median (IQR)	31 (17–43)	30 (17–44)
Age of onset (year), median (IQR)	1 (0–3)	
Disease duration (year), median (IQR)	28 (15–37)	
Sex, n (%)		
Female	41 (52)	18 (60)
Male	38 (48)	12 (40)
SMA type, n (%)		
1	7 (9)	
2	33 (42)	
3	39 (49)	
*SMN2* copy number, n (%)		
2	9 (16)	
3	31 (53)	
4+	18 (31)	
Unknown	21	
Weight (kg), median (IQR)	50 (33–65)	
Height (cm), median (IQR)	158 (145–170)	
BMI (kg/m^2^), median (IQR)	20.5 (16.1–23.4)	
Scoliosis, n (%)		
Present	50 (63)	
Not present	29 (37)	
Spondylodesis, n (%)		
Present	24 (30)	
Not present	55 (70)	
CT supported LP, n (%)		
Yes	44 (56)	
No	35 (44)	
Use of a-/traumatic needle, n (%)		
Traumatic	37 (47)	
Atraumatic	42 (53)	
Wheelchair-use, n (%)		
Never	9 (11)	
Occasionally	6 (8)	
Permanently	64 (81)	
Mobility, n (%)		
Never able to walk	40 (51)	
Lost ability to walk	24 (30)	
Still able to walk	15 (19)	
Ventilation-use, n (%)		
Never	55 (70)	
< 16 h	20 (25)	
> 16 h	4 (5)	
PEG/feeding tube, n (%)		
Yes	9 (11)	
No	70 (89)	

*IQR* interquartile range, *BMI* body mass index, *CT* computed tomography, *LP* lumbar puncture, *PEG* percutaneous endoscopic gastrostomy

### CHIT1 levels were elevated in SMA, but did not reflect disease severity

**Fig. 2:**
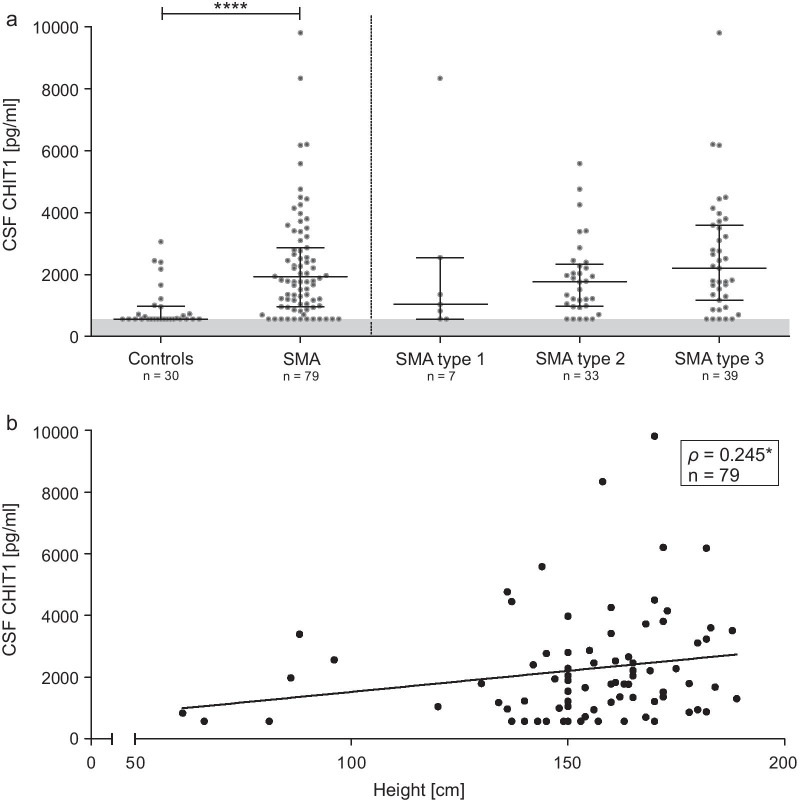
**a** Baseline analysis of CSF CHIT1 levels comparing diseased individuals to controls. Horizontal line shows median, whiskers illustrate interquartile range (0.25–0.75), each icon represents an individual patient, grey area marks range below the lower limit of quantification (563 pg/mL); ****p < 0.0001 calculated by Mann–Whitney U test. **b** Correlation between patients’ height and CSF CHIT1 levels before treatment initiation; each icon represents an individual patient; *p < 0.05 calculated by Spearman's rank-order correlation. CSF, cerebrospinal fluid; CHIT1, Chitotriosidase 1

CHIT1 levels in the CSF of patients with SMA before treatment initiation were elevated compared to the control group (MWU *p* < 0.0001; Fig. 2a, Table 2) and were associated with patients’ height (Fig. 2b). Of note, in 15% of the SMA CSF samples and 57% of the control CSF samples, CHIT1 was below the lower limit of quantification (LLOQ = 563 pg/mL).

**Table 2:** CSF CHIT1 levels in nusinersen-naïve SMA patients

	All SMA patients (n = 79)	Adult SMA patients (n = 58)	Pediatric SMA patients (n = 21)	Controls (n = 30)
CSF CHIT1 (pg/mL), median (IQR)	1787 (959–2866)	1838 (1035–3133)	1294 (883–2657)	563 (563–971)
Range	563–9810	563–9810	563–8341	563–3058
Below LLOQ, n (%)	12 (15)	8 (14)	4 (19)	17 (57)

*CHIT1* Chitotriosidase 1 concentration, *CSF* cerebrospinal fluid, *IQR* interquartile range, *LLOQ* lower limit of quantification (< 563 pg/mL)

CHIT1 levels in treatment-naïve patients were not associated with disease severity (as assessed by HFMSE, RULM, ALSFRS-R), baseline age, disease onset or disease duration after correction for patients’ height (see Additional file 2: Table S2).

CHIT1 levels and Qalb showed a weak correlation, but the significance of that correlation appeared to be caused by a single outlier. After exclusion of that outlier, no significant correlation between CHIT1 and Qalb could be detected, therefore we did not consider Qalb as a confounding factor regarding the analysis of CHIT1.

Before treatment initiation, CHIT1 levels did not differ significantly either regarding SMA type, *SMN2* copy number or between children and adults after adjustment for patients’ height (Fig. 2a). Moreover, no differences in CHIT1 levels between ambulatory and non-ambulatory patients could be detected.

### CHIT1 levels increased during nusinersen treatment

**Fig. 3:**
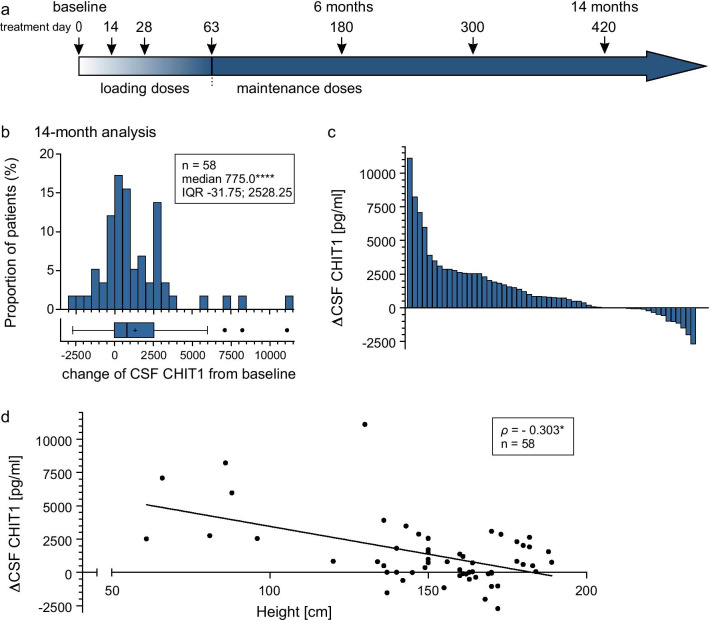
**a** Nusinersen dosing regimen according to the prescribing information. Arrows display time points of intrathecal nusinersen administration within the observation period. **b** Changes of CSF CHIT1 levels from baseline to 14 months, with each bar representing the proportion of patients related to the extent of CSF CHIT1 change. Box and whisker plots show median (vertical line), mean (+), interquartile range (boxes); individual points illustrate values outside of 1.5 × interquartile range (whiskers) from the median; ****p < 0.0001 calculated by Wilcoxon signed-rank test. **c** Change of CSF CHIT1 levels from baseline to 14 months with each bar representing a single patient. **d** Correlation between patients’ height and CSF CHIT1 change after 14 months of nusinersen treatment; each icon represents an individual patient; *p < 0.05 calculated by Spearman's rank-order correlation. ΔCHIT1, difference of Chitotriosidase 1 concentration (CHIT1) in cerebrospinal fluid (CSF) between baseline and 14-month follow-up

Within 14 months of nusinersen treatment, CHIT1 levels significantly increased (*p* < 0.0001; Table 3; Fig. 3b, c). In CSF of four patients, no change of CHIT1 levels was observed during the observational period because the amount was below the LLOQ.

**Table 3:** Dynamics in CSF CHIT1 levels during 14 months of nusinersen treatment

CSF CHIT1 (pg/mL)	14-month analysis				
	n	Median (IQR)	Difference versus baseline		*p* value
			Median (range)	(%)	
Overall	58	2963 (1726–4179)	775 (− 2713 to 11,103)	+ 43	< 0.0001****
Height < 131 cm	8	6370 (3328–9980)	4363 (826 to 11,103)	+ 309	0.011719*
Height > 131 cm	50	2759 (1699–4014)	545 (− 2713 to 3900)	+ 29	0.001747**
SMA type 1	6	2605 (1073–5731)	1675 (− 114 to 7086)	+ 180	n.s
SMA type 2	24	3026 (1744–4335)	897 (− 1154 to 11,103)	+ 51	0.000829***
SMA type 3	28	2847 (1710–4093)	543 (− 2713 to 3087)	+ 21	n.s
< 4 *SMN2* copies	30	3087 (1992–4528)	1066 (− 1536 to 8220)	+ 58	0.000960***
≥ 4 *SMN2* copies	13	2103 (1696–3789)	495 (− 2713 to 2303)	+ 22	n.s
Children	19	2909 (1860–5091)	984 (− 1536 to 8220)	+ 76	0.001592**
Adults	39	3016 (1702–4088)	495 (− 2713 to 11,103)	+ 24	0.007929**

*CHIT1* Chitotriosidase 1 concentration, *CSF* cerebrospinal fluid, *IQR* interquartile range, *n.s.* not significant

*p < 0.05; **p < 0.01; ***p < 0.001; ****p < 0.0001 calculated by Wilcoxon signed-rank test

Subgroup analyses are shown in Table 3, Additional file 3: Table S3 and Additional file 4: Fig. S1. After correction for patients' height, no differences in CHIT1 dynamics between subgroups regarding SMA type or *SMN2* copy number could be verified, but a strong inter-correlation was seen between the independent variables and patients' height. The CHIT1 increase was associated with patients’ height (*ρ* = − 0.303; *p* < 0.05; Fig. 3d) but did not correlate with age (*ρ* = − 0.231; n.s.). To determine which variable contributes most to the change of CHIT1 levels, we performed standard multiple regression considering patients’ height, age and *SMN2* copy number as independent variables. The regression model was statistically significant compared to the null model (F(3,39) = 4.443, *p* < 0.01) and explained 19.7% of the variance in CHIT1 dynamics. However, only height added statistically significantly to the prediction (*p* < 0.05) (Table 4).

**Table 4:** Impact of possible influencing variables on the change of CSF CHIT1 levels during 14 months

ΔCHIT1	B (CI95%)	SE B	β	R^2^	ΔR^2^
Model				0.255	0.197**
Constant	2.564** (1.046; 4.081)	0.750			
Patients’ height (cm)	− 0.014* (− 0.028; − 0.000)	0.007	− 0.449		
*SMN2* copy number	− 0.222 (− 0.694; 0.250)	0.233	− 0.166		
Age at start of therapy (year)	0.006 (− 0.015; 0.027)	0.010	0.110		

ΔCHIT1, difference of Chitotriosidase 1 concentration in CSF between baseline and 14-month follow-up

*p < 0.05; **p < 0.01

CHIT1 dynamics did not differ significantly between patients with an increase on HFMSE or CHOP score and patients who lost points on those motor scores within 14 months. Patients with no change on the scores were not included. No significant difference in CHIT1 dynamics was found regarding CT support or use of a traumatic puncture needle.

CSF levels of Qalb and total protein mildly but significantly increased during 14 months of nusinersen treatment, while CSF cell count did not change (Table 5).

**Table 5:** Changes of CSF routine parameters during 14 months of nusinersen treatment

CSF routine parameter	n	Baseline median (IQR)	14-month analysis median (IQR)	*p* value
CellCount (MPt/L)	57	1.0 (0.0–2.0)	1.0 (0.15–1.0)	n.s
Qalb (× 10^–3^)	56	4.1 (3.3–6.1)	4.7 (3.7–6.3)	0.000359***
Protein (mg/L)	58	308 (244–419)	345 (283–443)	0.000246***

*CSF* cerebrospinal fluid, *Qalb* ratio of serum/CSF albumin, *IQR* interquartile range, *n.s.* not significant

***p < 0.001 calculated by Wilcoxon signed-rank test

Within the routine CSF cytology, unusual monocytes or macrophages with indefinable inclusions emerged during nusinersen treatment (11 of 22 patients at least once within the cohort of the research site Dresden), whereas no inclusions were described in samples collected prior to treatment.

## Discussion

Our study has two remarkable results. First, CHIT1 levels were elevated in treatment-naïve SMA patients compared to controls and second, CHIT1 levels further increased following nusinersen treatment.

In SMA mouse models, motor neurons colocalized with an increased number of microglial cells, and reduced SMN protein levels were found to be related to increased microglial activation [31, 40]. Also, elevated levels of CHIT1 were associated with microglial cell activation in ALS mouse models and in patients with the occurrence of higher levels in fast-progressing ALS [20]. Increased levels of CHIT1 in patients with SMA might therefore reflect a general microglial activation and might illustrate the neuroinflammatory aspect in the pathogenesis of both, ALS and SMA. We did not observe associations either with patients’ age, SMA type or disease severity in SMA patients, which is in contrast to ALS. CHIT1 levels in SMA were lower than described for ALS [13, 15, 20–23, 41] or other neurodegenerative diseases [13, 16, 18, 19, 24, 29, 42, 43], implying a minor involvement of neuroinflammation in SMA and arguing against the usefulness of CHIT1 as a disease severity biomarker for SMA. However, it might be interesting to investigate adjunct anti-inflammatory treatment strategies in SMA [30, 44].

The observed increase of CHIT1 levels during nusinersen treatment was not associated with motor improvement and did not depend on disease severity but on patients’ height. Neither intrathecal infections nor influence of CT-guided procedure or type of lumbar puncture needle (traumatic vs. atraumatic) were observed in the study group, which argues against an administration-dependent influence on the increase of CHIT1 level during nusinersen treatment course. Contrary to our observations, Ando et al. [40] described a decrease of inflammatory and microglial activity in a SMA mouse model in response to treatment with a nusinersen-equivalent antisense oligonucleotide (ASO), which was assumed to account for a favorable effect of the therapy.

Therefore, the increase of CHIT1 during treatment course might be associated with an inflammatory process apart from the above discussed microglial activation in SMA disease. One could rather suspect the medication itself to contribute to the increasing CHIT1 levels. Nusinersen is administered intrathecally in periodic doses of 12 mg in 5 ml independently from patients' age or height. Because height was shown to be related to spinal CSF volume [45, 46], smaller individuals may attain higher drug levels relative to their CSF volume. In the course of treatment, we observed monocytic cells with indefinable, unspecified inclusions within the nusinersen-treated cohort of Dresden. Consistent with that, recently published research [47, 48] of two independent research groups reported the emergence of unusual macrophages with specific inclusions, which could be detected beginning from the second lumbar puncture and notably were not present before initiation of nusinersen treatment. These macrophages—labeled 'nusinophages'—were present in all investigated patients for at least one time during 14 months of nusinersen treatment and were not found in patients with repeated lumbar puncture without nusinersen administration, which leads to the assumption that the inclusions inside these macrophages may contain nusinersen or nusinersen metabolites. CHIT1 is not an exclusive marker of microglial cells, instead it can be secreted by different cells of the MPS. We therefore hypothesize that CHIT1 dynamics under nusinersen treatment may occur as a response of nusinersen-exposed circulating monocytes in the CSF. In addition, we found a mild increase of Qalb and total protein during therapy (fitting to [32, 33, 49]), which underline the occurrence of a low unspecific inflammatory reaction following treatment initiation.

Communicating hydrocephalus with unknown incidence and etiology following intrathecal administration of the ASO nusinersen in SMA patients [50] and after intrathecal ASO administration (tominersen) in Huntington’s Disease have been reported [51]. Whether the inclusions in CSF monocytes contain nusinersen, and which effect and relevance the stimulated intrathecal monocytes have on the occurrence of hydrocephalus, needs further investigation.

This study has some limitations. Most of the control samples (57%) and 15% of the SMA samples showed levels below the LLOQ of the measuring method, which results in a substantial floor effect. Furthermore, we did not test for genetic variants of the *CHIT1* gene (24 base pair duplication), which causes a chitotriosidase deficiency [11] and therefore could be partly responsible for the values below the LLOQ.

## Conclusion

To the best of our knowledge, this is the first study showing elevated CHIT1 concentrations in SMA patients. CHIT1 concentration is not useful to assess disease severity or to predict treatment response, but may indicate a certain role of (neuro)inflammation in the pathogenesis of SMA. During nusinersen treatment, increasing CHIT1 levels may indicate an immune response-like, off-target reaction. Whether this observation is limited to nusinersen or represents a general reaction to intrathecal ASO administration, needs to be further evaluated, since it is an establishing approach in the treatment of neurodegenerative diseases [51–54].

## Supplementary Information

**Additional file 1: Table S1.** Subgroup characteristics. *IQR* interquartile range.

**Additional file 2: Table S2.** Correlation between CSF CHIT1 level and age/disease severity scores in nusinersen-naïve patients with SMA. *CHIT1* Chitotriosidase 1 concentration, *CSF* cerebrospinal fluid, *HFMSE* Hammersmith functional. Motor Scale Expanded; *RULM* Revised upper limb module, *ALSFRS-R* revised ALS functional. Rating Scale; *n.s.* not significant, *ρ* partial rank correlation coefficient corrected for patients’ height.

**Additional file 3: Table S3.** Changes in CHIT1 levels during the observation period of 14 months regarding different subgroups. *CHIT1* Chitotriosidase 1 concentration, *CSF* cerebrospinal fluid, *IQR* interquartile range, *n.s.* not significant. *p < 0.05; **p < 0.01 calculated by Friedman test with post-hoc Dunn–Bonferroni adjustment after listwise exclusion of data.

**Additional file 4: Fig. S1.** (**a**–**c**) Intraindividual CHIT1 dynamics regarding 3 time points within 14 months of nusinersen treatment referring to SMA subtype. Light grey box indicates normal range determined by upper level of confidence interval (95%) calculated from controls; darker grey area marks range below the lower limit of quantification. Dashed black line indicates the trajectory of CHIT1 median. For details see Additional file 3: Table S3. CSF, cerebrospinal fluid; CHIT1, Chitotriosidase 1; *p < 0.05 calculated by Friedman test with post-hoc Bonferroni adjustment n = 54 (**d**) Differences in height between different clinical subtypes. ****p < 0.0001 calculated by Kruskal–Wallis-test including patients shown in (**a**–**c**, **e**, **f**). (**e**, **f**) Intraindividual CHIT1 dynamics regarding 3 time points within 14 months of nusinersen treatment referring to patients' height. Light grey box indicates normal range determined by upper level of confidence interval (95%) calculated from controls; darker grey area marks range below the lower limit of quantification. Dashed black line indicates the trajectory of CHIT1 median. For details see Additional file 3: Table S3 CSF, cerebrospinal fluid; CHIT1, Chitotriosidase 1 concentration; *p < 0.05; **p < 0.01 calculated by Friedman test with post-hoc Dunn–Bonferroni adjustment n = 54.

## Data Availability

The datasets used and/or analyzed during the current study are available from the corresponding author on reasonable request.
