# Supplementary material for: Increased chitotriosidase 1 concentration following nusinersen treatment in spinal muscular atrophy
Source: Orphanet J Rare Dis. 2021 Jul 28;16:330. doi: 10.1186/s13023-021-01961-8 (PMC8320162; doi:10.1186/s13023-021-01961-8)
Supplement: Supplementary file 1 — Additional file 1: Table S1. Subgroup characteristics. IQR interquartile range. [file 13023_2021_1961_MOESM1_ESM.docx]

**Additional table 1** Subgroup characteristics

| BASELINE |  | Pediatric patients  (n=21) | Adult patients  (n=58) |
| --- | --- | --- | --- |
| Age [yr],  median (IQR) |  | 11  (3 - 15) | 33  (29 - 47) |
| Age of onset [yr],  median (IQR) |  | 0  (0 - 1) | 2  (1 - 7) |
| Disease duration [yr],  median (IQR) |  | 10  (3 - 14) | 31  (24 - 44) |
| Sex,  n (%) | female  male | 11 (52)  10 (48) | 30 (52)  28 (48) |
| SMA type,  n (%) | 1  2  3 | 7 (33)  10 (48)  4 (19) | -  23 (40)  35 (60) |
| *SMN2* copy number,  n (%) | 2  3  4+  n.d. | 5 (46)  5 (46)  1 (9)  10 | 4 (9)  26 (55)  17 (36)  11 |
| Weight [kg],  median (IQR) |  | 25  (15 - 40) | 61  (44 - 70) |
| Height [cm],  median (IQR) |  | 137  (92 - 155) | 164  (150 - 172) |

IQR, interquartile range
