# Supplementary material for: Increased chitotriosidase 1 concentration following nusinersen treatment in spinal muscular atrophy
Source: Orphanet J Rare Dis. 2021 Jul 28;16:330. doi: 10.1186/s13023-021-01961-8 (PMC8320162; doi:10.1186/s13023-021-01961-8)
Supplement: Supplementary file 2 — Additional file 2: Table S2. Correlation between CSF CHIT1 level and age/disease severity scores in nusinersen-naïve patients with SMA. CHIT1 Chitotriosidase 1 concentration, CSF cerebrospinal fluid, HFMSE Hammersmith functional. Motor Scale Expanded; RULM Revised upper limb module, ALSFRS-R revised ALS functional. Rating Scale; n.s. not significant, ρ partial rank correlation coefficient corrected for patients’ height. [file 13023_2021_1961_MOESM2_ESM.docx]

**Additional table 2** Correlation between CSF CHIT1 level and age / disease severity scores in nusinersen-naïve patients with SMA

|  | CSF CHIT1 [pg/mL] |
| --- | --- |
| Age | *ρ* = 0.037  n.s.  n = 79 |
| Disease onset | *ρ* = - 0.099  n.s.  n = 79 |
| Disease duration | *ρ* = 0.072  n.s.  n = 79 |
| HFMSE | *ρ* = - 0.009  n.s.  n = 63 |
| RULM | *ρ* = - 0.071  n.s.  n = 65 |
| ALSFRS-R | *ρ* = - 0.096  n.s.  n = 66 |

CHIT1, Chitotriosidase 1 concentration; CSF, cerebrospinal fluid; HFMSE, Hammersmith Functional

Motor Scale Expanded; RULM, Revised Upper Limb Module; ALSFRS-R, revised ALS Functional

Rating Scale; n.s., not significant; *ρ*, partial rank correlation coefficient corrected for patients’ height
