# Supplementary material for: Increased chitotriosidase 1 concentration following nusinersen treatment in spinal muscular atrophy
Source: Orphanet J Rare Dis. 2021 Jul 28;16:330. doi: 10.1186/s13023-021-01961-8 (PMC8320162; doi:10.1186/s13023-021-01961-8)
Supplement: Supplementary file 3 — Additional file 3: Table S3. Changes in CHIT1 levels during the observation period of 14 months regarding different subgroups. CHIT1 Chitotriosidase 1 concentration, CSF cerebrospinal fluid, IQR interquartile range, n.s. not significant. *p < 0.05; **p < 0.01 calculated by Friedman test with post-hoc Dunn–Bonferroni adjustment after listwise exclusion of data. [file 13023_2021_1961_MOESM3_ESM.docx]

**Additional table 3** Changes in CHIT1 concentration during the observation period of 14 months regarding different subgroups

|  | n | Baseline | | 6-month analysis | | 14-month analysis | |  |
| --- | --- | --- | --- | --- | --- | --- | --- | --- |
|  |  | median  (IQR) | | median  (IQR) | p value | median  (IQR) | p value |  |
| CSF CHIT1  [pg/mL] | 54 | 1853  (1046 - 3267) | | 2786  (1206 - 3330) | n.s. | 2963  (1732 - 4179) | 0.001111** |  |
| SMA type 1 | 5 | 1034  (695 - 1954) | | 1192  (1004 - 4112) | n.s. | 3350  (1552 - 6370) | n.s. |  |
| SMA type 2 | 22 | 1836  (1143 - 2514) | | 2786  (1703 - 3095) | n.s. | 3168  (1772 - 4528) | 0.012536* |  |
| SMA type 3 | 27 | 2517  (1294 - 3803) | | 2883  (1147 - 3846) | n.s. | 2784  (1702 - 4088) | n.s. |  |
| < 131cm | 8 | | 1411  (629 - 2405) | 3124  (1344 - 3574) | n.s | 6370  (3328 - 9980) | 0.001396** | |
| > 131cm | 46 | | 1913  (1197 - 3457) | 2604  (1206 - 3123) | n.s. | 2759  (1710 - 4014) | 0.049466* | |

CHIT1, Chitotriosidase 1 concentration; CSF, cerebrospinal fluid; IQR, interquartile range; n.s., not

significant; *, p < 0.05; **, p < 0.01 calculated by Friedman test with post-hoc Dunn-Bonferroni adjustment after listwise exclusion of data
